# Modulation of the Gut–Lung Axis by Water Kefir and Kefiran and Their Impact on Toll-like Receptor 3-Mediated Respiratory Immunity

**DOI:** 10.3390/biom14111457

**Published:** 2024-11-17

**Authors:** Stefania Dentice Maidana, Julio Nicolás Argañaraz Aybar, Leonardo Albarracin, Yoshiya Imamura, Luciano Arellano-Arriagada, Fu Namai, Yoshihito Suda, Keita Nishiyama, Julio Villena, Haruki Kitazawa

**Affiliations:** 1 Laboratory of Immunobiotechnology, Reference Centre for Lactobacilli (CERELA-CONICET), San Miguel de Tucumán 4000, Argentina; stefi.dentice@gmail.com (S.D.M.); lalbarracin@herrera.unt.edu.ar (L.A.); 2 Cátedra de Inmunología, Instituto de Microbiología, Facultad de Bioquímica, Química y Farmacia, Universidad Nacional de Tucumán, San Miguel de Tucumán 4000, Argentina; nic0laz@hotmail.com; 3 Food and Feed Immunology Group, Laboratory of Animal Food Function, Graduate School of Agricultural Science, Tohoku University, Sendai 980-8572, Japan; yoshiya.imamura.p8@dc.tohoku.ac.jp (Y.I.); luciarellano1996@gmail.com (L.A.-A.); fu.namai.a3@tohoku.ac.jp (F.N.); keita.nishiyama.a6@tohoku.ac.jp (K.N.); 4 Livestock Immunology Unit, International Education and Research Centre for Food and Agricultural Immunology (CFAI), Graduate School of Agricultural Science, Tohoku University, Sendai 980-8572, Japan; 5 Department of Food, Agriculture and Environment, Miyagi University, Sendai 980-8572, Japan; suda@myu.ac.jp

**Keywords:** water kefir, respiratory immunity, gut–lung axis, antiviral immunity, TLR3, probiotics

## Abstract

The beneficial effect of milk kefir on respiratory heath has been previously demonstrated; however, water kefir and kefiran in the context of respiratory viral infections have not been investigated. Water kefir and kefiran could be alternatives to milk kefir for their application in persons with lactose intolerance or milk allergy and could be incorporated into vegan diets. Using mice models, this work demonstrated that the oral administration of water kefir or kefiran can modulate the respiratory Toll-like receptor (TLR3)-mediated innate antiviral immunity and improve the resistance to respiratory syncytial virus (RSV) infection. The treatment of mice with water kefir or kefiran for 6 days improved the production of interferons (IFN-β and IFN-γ) and antiviral factors (Mx2, OAS1, RNAseL, and IFITM3) in the respiratory tract after the activation of the TLR3 signaling pathway, differentially modulated the balance of pro- and anti-inflammatory cytokines, reduced RSV replication, and diminished lung tissue damage. Maintaining a proper balance between anti-inflammatory and pro-inflammatory mediators is vital for ensuring an effective and safe antiviral immune response, and the results of this work show that water kefir and kefiran would help to maintain that balance promoting a controlled inflammatory response that defends against infection while minimizing tissue damage.

## 1. Introduction

Kefir is a traditional fermented beverage obtained by the fermentation of milk (milk kefir) or water-sugar (water kefir) with kefir grains, which contain a complex mixture of lactic acid bacteria (LAB), acetic bacteria, and yeasts. The final product is a beverage composed of diverse microorganisms growing mainly in the grains and a fluid matrix containing exopolysaccharides (EPSs), organic acids, lipids, proteins, and peptides [1,2]. In recent years, there has been an increase in kefir consumption because of its association with beneficial effects on health. Regular intake of kefir has been linked to antihypertensive and anticarcinogenic effects, the decline of inflammatory bowel disease severity, and several benefits related to metabolic disorders including enhanced insulin sensitivity and improved lipid profile [1,2]. Those positive effects on health have been associated with the ability of kefir to influence the host’s antioxidant capacity, the immune system and the intestinal microbiota. In addition, there are studies that indicate that kefir feeding could modulate the gut–lung axis and thus exert beneficial effects on respiratory health [3,4,5,6]. Kefiran produced by *Lactobacillus kefiranofaciens* in kefir grains is an EPS having a repeating structure with glucose and galactose residues in the chain sequence and has been suggested to exert many health-promoting effects of kefir [1,2].

It was shown that both milk kefir and pasteurized milk kefir when orally administered to adult BALB/c mice for 5 days increased the number of immunoglobulin A (IgA)^+^ cells in the respiratory tract as well as the phagocytic activity of pulmonary macrophages [3]. Further studies suggested that the kefiran produced by the kefir bacterium *L. kefiranofaciens* [1,2], would be a key molecule involved in the modulation of the gut–lung axis. Kefiran produced by *L. kefiranofaciens* subsp. *kefiranofaciens* ATCC 43761 and administered by the oral route to adult mice for 7 days was able to reduce the systemic inflammatory response induced by the intraperitoneal challenge with lipopolysaccharide (LPS) [7]. Orally administered kefiran not only reduced the activation of nuclear factor κB (NF-κB) pathway in the gut and the levels of serum interleukin 6 (IL-6), but it also diminished NF-κB activation in the lungs, indicating its ability to modulate immunity in the respiratory tract [7].

The effect of milk kefir in the respiratory tract was also tested in models of lung chronic inflammation like allergy and pulmonary fibrosis. It was shown that the administration of milk kefir to adult BALB/c mice sensitized and nasally challenged with ovalbumin significantly reduced allergen-induced airway hyper-responsiveness [8]. Milk kefir-treated mice had lower levels of respiratory inflammatory cells, particularly eosinophils, as well as IgE, IL-4, and IL-13. Similarly, adult BALB/c mice sensitized with ovalbumin and then treated with kefiran before the induction of airway hyperresponsiveness showed lower eosinophil counts, mucus production, and IL-4 and IL-5 concentrations in the respiratory tract [4]. In a model of bleomycin-induced pulmonary fibrosis in adult Wistar rats, it was shown that oral treatment with kefir made with goat milk was able to reduce serum transforming growth factor β1 (TGF-β1), increase interferon γ (IFN-γ), and reduce the expression of alpha-smooth muscle actin (α-SMA) in lung myofibroblasts [5]. Interestingly, it was recently demonstrated that kefir peptides, generated by the kefir grain fermentation of milk proteins, were able to diminish the lung injury induced by the intratracheal injection of bleomycin [9]. In this work, adult C57BL/6 J mice were orally administered kefir peptides for 4 days before the challenge with bleomycin. The treatment prevented body weight loss, reduced the levels of the serum indicator of lung fibrosis MMP7, and diminished the alterations of lung architecture. Of note, kefir peptide-treated mice had improved expression levels of the antioxidant factors Nrf2, Nqo1, Cat, Sod1, and Ho-1 in lungs as well as reduced expressions of IL-6, inducible nitric oxide synthase (iNOS), chemokine (C-X-C motif) ligand 2 (CXCL2), IL-4, IL-1β, and tumor necrosis factor α (TNF-α) [9]. In line with this study, in a model of particulate matter-induced lung inflammation in transgenic homozygous NF-κB-luciferase^+/+^ mice, it was shown that kefir peptide administration exerted anti-inflammatory and antioxidant protective effects [6].

Although these studies indicate that kefir and kefiran may have positive effects on respiratory health, scare studies have been conducted to investigate its potential protective effect in the context of respiratory viral infections. In a double-blind randomized controlled trial conducted with 100 patients with confirmed severe acute respiratory syndrome coronavirus 2 (SARS-CoV-2) infection, milk kefir was administered for two weeks starting from the day that the patients went to the hospital for medical treatment. Several clinical outcomes including fever, pain, gastrointestinal symptoms, dyspnea, and inflammatory indices were determined at the beginning and end of the study [10]. Significantly lower and higher blood neutrophils and lymphocytes counts, respectively, were found in milk kefir-treated patients compared to the control group. In addition, oxygen saturation was improved in the milk kefir group compared to control patients [10]. To the best of our knowledge, no other study evaluated the effect of kefir in the modulation of respiratory antiviral immunity. Furthermore, studies mainly focused on milk kefir, while the immunomodulatory properties of water kefir have not been investigated.

We recently demonstrated that water kefir administration can modulate the intestinal innate antiviral immune response triggered by the activation of the pattern recognition receptor (PRR) Toll-like receptor 3 (TLR3) [11]. Kefir-treated mice had significantly lower levels of intestinal inflammatory factors TNF-α, IL-15, IL-6, RAE-1, and NKG2D, and improved levels of IFN-β and IFN-γ, as well as the regulatory cytokine IL-10. This differential cytokine profile induced by water kefir administration was associated with a significant reduction in inflammatory-mediated intestinal damage. Considering these results and the studies mentioned above, we aimed to evaluate the potential modulatory effects of water kefir and kefiran, when orally administered, on the respiratory innate antiviral immunity. For this purpose, mice were fed water kefir or kefiran and then challenged nasally with the TLR3 agonist poly(I:C) or infected with respiratory syncytial virus (RSV). The respiratory innate antiviral immune response, lung damage and the resistance to the viral infection were evaluated.

## 2. Materials and Methods

### 2.1. Water Kefir and Kefiran Samples

The water kefir was prepared as described previously [11]. Briefly, kefir grains and mascabo sugar were added to sterile water (5% *w*/*v*), and the fermentation was performed at room temperature in plastic bottles for 24 h. Kefiran was extracted following the method described previously by Medrano et al. [12]. A measured amount of kefir grains was treated with boiling water in a 1:10 ratio (*w*/*w*) for 3 h with intermittent stirring. The mixture was then centrifuged at 10,000× *g* for 20 min at 20 °C using a Sorvall RC-5B Plus centrifuge (Hunan, China). The polysaccharide in the supernatant was precipitated by adding two volumes of cold ethanol, and the solution was stored at −20 °C overnight. Then, the mixture was centrifuged at 10,000× *g* for 20 min at 4 °C. The resulting pellets were dissolved in hot water, and the precipitation process was repeated once. Finally, the polysaccharide was dissolved in hot distilled water to form a kefiran solution, which was subsequently lyophilized (Heto FD4, Heto-Holten, Allerød, Denmark).

The concentration of the polysaccharide was determined using the anthrone method with glucose as a standard (Sigma, St. Louis, MO, USA) [12]. All samples were checked for the absence of free sugars using qualitative thin-layer chromatography (TLC) on Silica gel G-type 60 plates (Merck, Darmstadt, Germany) with a mobile phase of n-propanol-acetic acid–water (70:20:10 *v*/*v*/*v*). TLC plates were developed using p-amino benzoic acid (7 g/L) and o-phosphoric acid (30 g/L) in methanol. The absence of proteins in the kefiran solution was confirmed using the Bradford method (Sigma, St. Louis, MO, USA). Kefiran solutions were prepared by dissolving lyophilized kefiran in PBS, and the solutions were sterilized by filtration through a 0.45 μm pore filter before use in experiments.

### 2.2. Animals and Feeding Procedures

Male 5-week-old BALB/c mice were sourced from the closed colony maintained at CERELA-CONICET (San Miguel de Tucumán, Argentina) and housed in plastic cages at room temperature. The experiments involved groups of 5 mice per parameter studied. Water kefir and kefiran were orally administered to separate groups of mice. Water kefir was administered in a dilution of 1:5 for 6 consecutive days, according to our previous publication [11]. Kefiran was administered for 6 consecutive days in a dose of 0.75 mg per day in the drinking water according to previous works [7,13]. Mice were deprived of water for 4 h prior to the administration of water kefir or kefiran. Control groups received only water. Throughout the study, all mice were fed a balanced diet ad libitum. The research was conducted in strict compliance with the Guide for Care and Use of Laboratory Animals and was approved by CERELA’s Ethical Committee under the protocol CRL-CICUAL-IBT-2024/3A. Special care was taken to minimize animal suffering, and no discomfort or premature deaths were observed during the study.

### 2.3. Intranasal Administration of Poly(I:C) and RSV Infection

Two days after the kefir and kefiran treatments, mice were administered poly(I:C) (Sigma-Aldrich, St. Louis, MO, USA), a TLR3 agonist. A solution of 250 μg poly(I:C) (equivalent to 10 mg/kg body weight) in 100 μL of PBS was applied dropwise to the nares of the mice to induce TLR3-mediated lung inflammation [14,15]. Control animals received 100 μL of PBS. Poly(I:C) administration was carried out in three doses with a 24 h rest between each dose.

The human RSV strain A2 was propagated in Vero cells following previously established methods [14,15]. Vero cells were cultured in Dulbecco’s Modified Eagle Medium (DMEM) and infected with RSV at a multiplicity of infection (MOI) of 1 in a 5 mL volume for 3 h at 37 °C and 5% CO₂. After the infection, 7 mL of DMEM with 10% fetal bovine serum (FBS), 0.1% penicillin-streptomycin, and 0.001% ciprofloxacin were added, and the cells were incubated until extensive syncytium formation was observed. Cells were then scraped and sonicated, and the debris was cleared by centrifugation at 700× *g* for 10 min at 4 °C. The viral supernatant was purified using a sucrose density gradient and stored in 30% sucrose at −80 °C.

For in vivo infection, mice were challenged with 10^6^ plaque-forming units (PFUs) of RSV delivered nasally one day after the last water kefir or kefiran administrations. Lung RSV titers and tissue damage were assessed two days after infection. RSV titers were determined using an immunoplaque assay as previously described [14,15]. Lung tissue was homogenized, clarified, and added to Vero cell monolayers in triplicate. After a 3 h incubation, fresh medium (DMEM with 10% FBS, 0.1% Pen-Strep, and 0.001% ciprofloxacin) replaced the supernatants. Once extensive syncytia appeared, the monolayers were fixed with a cold acetone–methanol solution (60:40). RSV-specific antibodies (anti-F and anti-G) were used, followed by horseradish peroxidase-conjugated secondary antibodies. Plaques were visualized using a DAB substrate kit (Sigma-Aldrich, St. Louis, MO, USA), and the results were expressed as log_10_ PFU/g of lung tissue.

### 2.4. Lung Injury Parameters

Broncho-alveolar lavage (BAL) samples were collected as previously outlined [14]. In brief, the trachea was surgically exposed and intubated using a catheter, followed by two sequential lavages with sterile PBS for each mouse. The recovered fluid was centrifuged at 900× *g* for 10 min and then stored at −70 °C for later analysis.

To assess lung injury, protein and albumin levels, which indicate increased permeability of the broncho-alveolar–capillary barrier, and lactate dehydrogenase (LDH) activity, a marker of general cytotoxicity, were measured in the cell-free BAL fluid. Albumin concentration was determined using a colorimetric method based on albumin’s binding to bromocresol green, employing a diagnostic kit from Wiener Lab (Buenos Aires, Argentina). Protein content was measured by the bicinchoninic (BCA) protein assay (Pierce Biotechnology Inc., Rockford, IL, USA). LDH activity, expressed in units per liter of BAL fluid, was quantified by measuring the production of NADH (the reduced form of nicotinamide adenine dinucleotide) following Wiener Lab’s standard reagents and procedures. Histopathological examination was also performed to further evaluate tissue damage. Lungs were fixed in 4% formalin and embedded in histowax (Leica Microsystems, Wetzlard, Germany). Histopathological assessment was performed on 5 µm tissue sections stained with hematoxylin–eosin.

### 2.5. Leukocyte Counts in BAL

The total leukocyte counts in BAL samples were determined using a hemocytometer, while differential cell counts were carried out by staining blood smears with May-Grünwald-Giemsa stain and counting 200 cells per smear [15].

### 2.6. Cytokine Concentrations in BAL

BAL samples were stored at −70 °C for cytokine analysis. Tumor necrosis factor (TNF)-α, IFN-γ, IFN-β, IL-6, IL-10, IL-27, MCP-1, and KC levels in BAL fluid were determined using commercially available ELISA kits, following the manufacturer’s instructions (R&D Systems, Minneapolis, MN, USA).

### 2.7. Alveolar Macrophage Primary Cultures

Primary cultures of murine alveolar macrophages were established following previously described protocols [15]. Briefly, macrophages were harvested from mice through BAL sampling, using 1 mL of warm sterile PBS containing 5 mM EDTA. The cells were transferred to sterile tubes, washed twice with PBS, and resuspended in RPMI 1640 medium supplemented with 10% FBS, 1 mM L-glutamine, and 100 U/mL penicillin-streptomycin. The BAL cells were plated in 24-well plates at a density of 10^5^ cells per well and incubated for 2 h at 37 °C in a 5% CO₂ atmosphere to allow cell adherence. After non-adherent cells were removed, macrophages were cultured in RPMI 1640 with 10% FBS, 1 mM L-glutamine, and 100 U/mL penicillin-streptomycin at 37 °C with 5% CO_2_ for 24 h prior to stimulation. Alveolar macrophages were stimulated with poly(I:C) (50 μg/mL), and mRNA was extracted 12 h after to evaluate the gene expression of cytokines and antiviral factors.

### 2.8. RT-PCR

A two-step real-time quantitative PCR was conducted to analyze the expression of IFN-β, IFN-γ, and the antiviral factors IFN-induced GTP-binding protein Mx2, 2′-5′-oligoadenylate synthetase 1 (OAS1), ribonuclease L (RNAseL), and IFN-induced transmembrane protein 3 (IFITM3) in alveolar macrophage cultures [15]. Total RNA was extracted using TRIzol reagent (Invitrogen, Waltham, Massachusetts, USA) following the manufacturer’s instructions, while cDNA synthesis was performed using a Quantitect reverse transcription kit (Qiagen, Tokyo, Japan). The real-time PCR was performed on a 7300 real-time PCR system (Applied Biosystems, Warrington, UK) using the Platinum SYBR Green qPCR SuperMix with uracil-DNA glycosylase (UDG) and 6-carboxyl-X-rhodamine (ROX) (Invitrogen). Primers used were described previously [15].

The PCR cycling conditions were as follows: 2 min at 50 °C, 2 min at 95 °C, followed by 40 cycles of 15 s at 95 °C, 30 s at 60 °C, and 30 s at 72 °C. Expression levels were normalized using β-actin as a reference to account for variations in total cDNA among samples.

### 2.9. Statistical Analysis

All experiments were conducted in triplicate, and the data were presented as mean ± standard deviation (SD). The normal distribution of the data was verified before applying a two-way ANOVA for statistical analysis. To compare the means between groups, Tukey’s post hoc test was used. Statistical significance was determined at *p*-values less than 0.05.

## 3. Results

### 3.1. Water Kefir and Kefiran Reduce Lung Tissue Injuries Induced by Poly(I:C)

**Figure 1:**
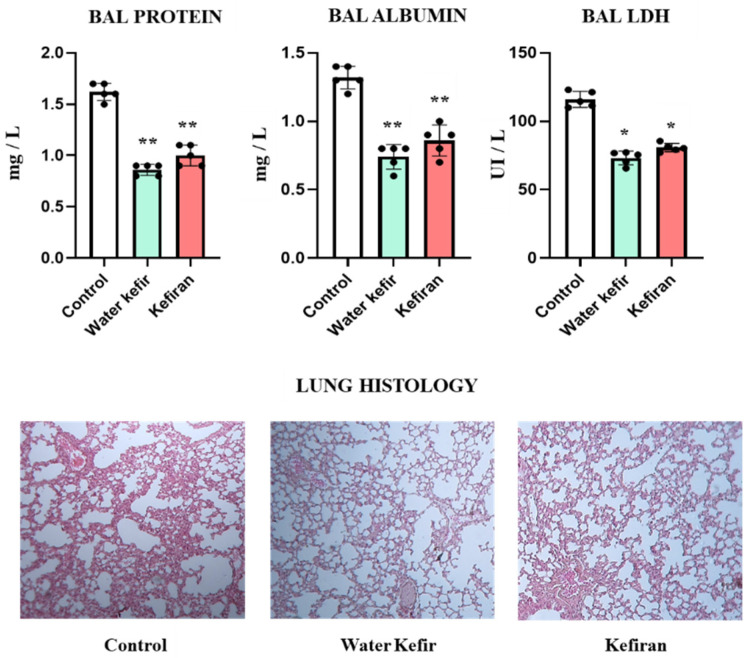
Effect of water kefir and kefiran on TLR3-mediated lung damage. Mice were fed water kefir or kefiran for 6 days and stimulated on days 7, 8, and 9 with the TLR3 agonist poly(I:C) by the nasal route. Mice without water kefir or kefiran treatment and stimulated with poly(I:C) were used as control. The concentrations of broncho-alveolar lavage (BAL) proteins and albumin, the activity of BAL lactate dehydrogenase (LDH), and lung histology were determined 2 days after TLR3 activation. Hematoxylin–eosin stain of histological slices of lung micrographs at 10× are shown. The results are expressed as mean ± SD. Significant differences were shown compared to the poly(I:C)-treated control group at *p* < 0.05 (*) or *p* < 0.01 (**).

We first aimed to evaluate whether orally administered water kefir or kefiran had the capacity to modulate the injury induced by poly(I:C) in the respiratory tract. Then, mice were treated with water kefir or kefiran and then challenged with the TLR3 agonist. The concentrations of proteins and albumin and the levels of LDH were determined in BAL samples two days after the last poly(I:C) administration to measure lung damage (Figure 1). As reported previously [15], nasally administered poly(I:C) significantly augmented the levels of the three biochemical markers of lung injury (basal levels of BAL proteins and LDH are approximately 0.4 mg/L and 18 UI/L, respectively, while BAL albumin is under the detection levels). Of note, animals treated with water kefir or kefiran had significantly lower levels of BAL protein, albumin, and LDH than controls, and with no differences between them (Figure 1).

In addition, histological examinations of lung samples were performed to confirm the effect of water kefir and kefiran in the protection against severe inflammatory damage. In control mice, an alteration of the pulmonary architecture was observed, characterized by an extensive infiltration of inflammatory cells, thickening of the interstitial tissue, and reduction in the air spaces. These changes were considerably milder in the mice treated with water kefir or kefiran (Figure 1).

### 3.2. Water Kefir and Kefiran Modulate the Respiratory Innate Immune Response Triggered by Poly(I:C)

**Figure 2:**
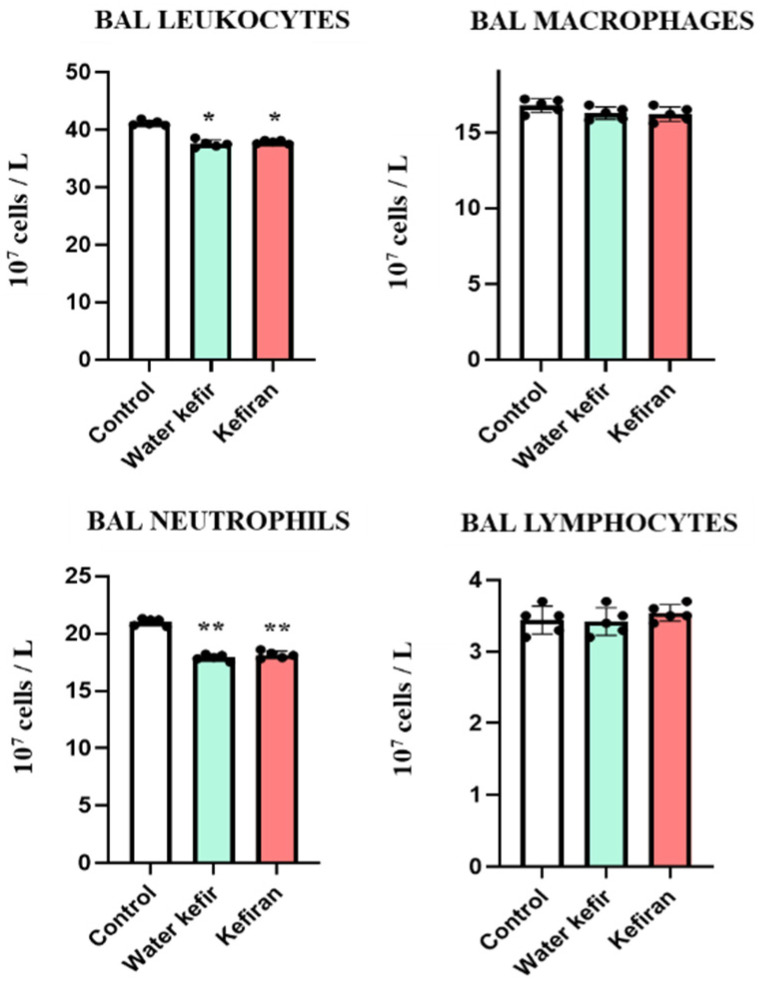
Effect of water kefir and kefiran on TLR3-mediated lung inflammatory cells infiltration. Mice were fed water kefir or kefiran for 6 days and stimulated on days 7, 8 and 9 with the TLR3 agonist poly(I:C) by the nasal route. Mice without water kefir or kefiran treatment and stimulated with poly(I:C) were used as control. The numbers of broncho-alveolar lavage (BAL) leukocytes, macrophages, neutrophils, and lymphocytes were determined 2 days after TLR3 activation. The results are expressed as mean ± SD. Significant differences were shown compared to the poly(I:C)-treated control group at *p* < 0.05 (*) or *p* < 0.01 (**).

We next aimed to assess whether the oral treatments with water kefir or kefiran differentially modulated the respiratory innate immune response triggered by poly(I:C). Then, the levels of immune cell populations in BAL samples were determined (Figure 2). In basal conditions, BAL immune cells contain mainly alveolar macrophages (basal levels of BAL leukocytes, macrophages, and lymphocytes are approximately 6.7, 5.9, and 0.8 × 10^7^ cells/L, respectively, while neutrophils are not detected) [15,16]. The challenge with poly(I:C) significantly increased the numbers of BAL leukocytes, macrophages, and lymphocytes and induced the infiltration of neutrophils (Figure 2). Mice treated with water kefir and kefiran had lower counts of BAL leukocytes than controls, an effect that was mainly explained by the neutrophils counts since there were no differences in the numbers of BAL macrophages and lymphocytes between the treated and control animals (Figure 2).

**Figure 3:**
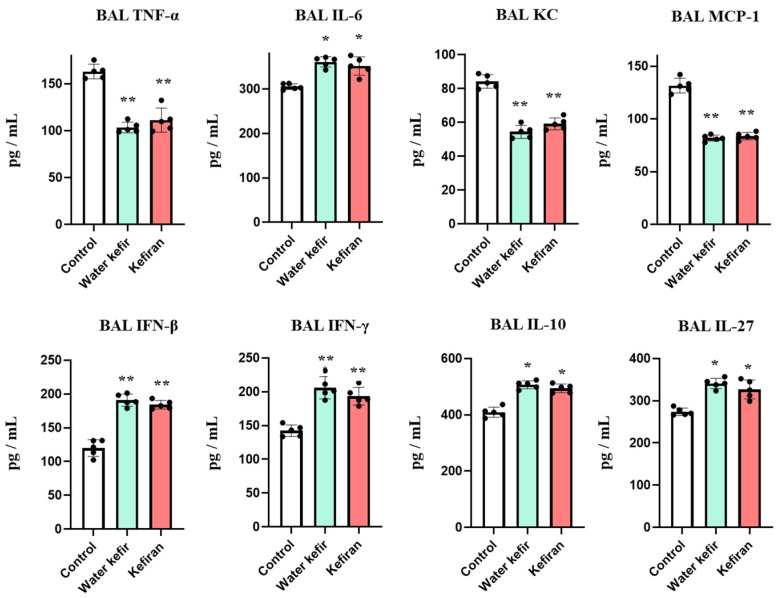
Effect of water kefir and kefiran on TLR3-mediated lung cytokine response. Mice were fed water kefir or kefiran for 6 days and stimulated on days 7, 8, and 9 with the TLR3 agonist poly(I:C) by the nasal route. Mice without water kefir or kefiran treatment and stimulated with poly(I:C) were used as control. The concentrations of broncho-alveolar lavage (BAL) TNF-α, IL-6, KC, MCP-1, IFN-β, IFN-γ, IL-10, and IL-27 were determined 2 days after TLR3 activation. The results are expressed as mean ± SD. Significant differences were shown compared to the poly(I:C)-treated control group at *p* < 0.05 (*) or *p* < 0.01 (**).

The levels of inflammatory cytokines and chemokines, IFNs, and regulatory cytokines were also determined in BAL samples (Figure 3). Basal levels of TNF-α, IL-6, KC, and MCP-1 in BAL samples are approximately 60, 150, 20, and 30 pg/mL, respectively [15,16]. The nasal administration of poly(I:C) increased the levels of the four inflammatory factors in BAL samples. However, mice treated with water kefir or kefiran had significantly lower levels of TNF-α, KC, and MCP-1 and higher concentrations of IL-6 than controls (Figure 3). Poly(I:C) also augmented the levels of BAL IFN-β and IFN-γ (basal concentrations of these IFNs are approximately 60 and 50 pg/mL, respectively). Water kefir- and kefiran-treated animals had significantly higher levels of IFNs than controls. Similarly, the activation of TLR3 in the respiratory tract increased the levels of the regulatory cytokines IL-10 and IL-27 (basal levels of BAL IL-10 and IL-27 are approximately 110 and 150 pg/mL, respectively) and the treatments with water kefir and kefiran significantly augmented the levels of both cytokines (Figure 3). Of note, when the profiles of cytokines in the respiratory tract after poly(I:C) administration were compared between water kefir- and kefiran-treated animals, no differences were detected.

**Figure 4:**
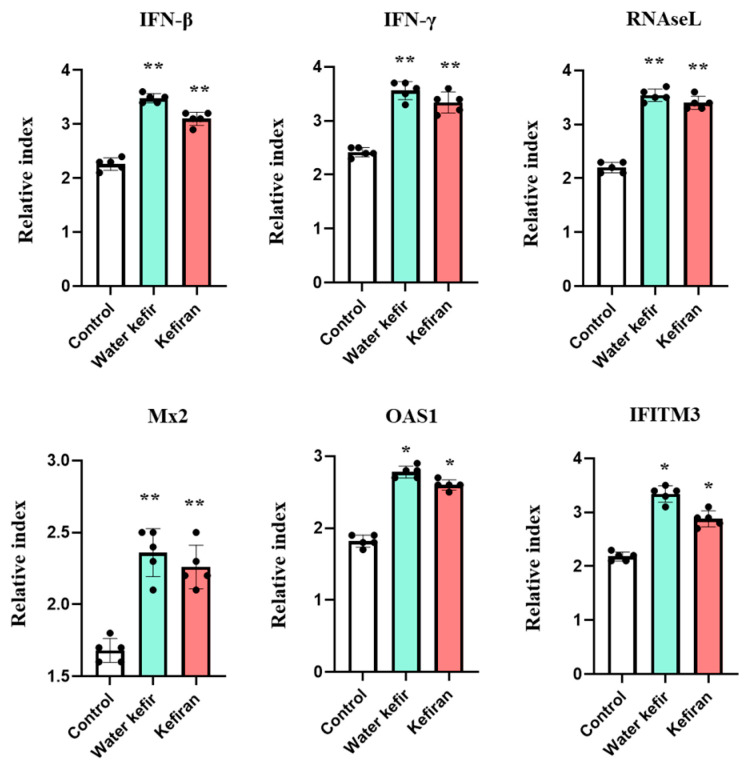
Effect of water kefir and kefiran on TLR3-mediated alveolar macrophages antiviral factors response. Mice were fed water kefir or kefiran for 6 days; on day 7, alveolar macrophages were collected and stimulated in vitro with the TLR3 agonist poly(I:C). Alveolar macrophages obtained from mice without water kefir or kefiran treatment and stimulated in vitro with poly(I:C) were used as control. The expressions of IFN-β, IFN-γ, Mx2, OAS1, RNAseL, and IFITM3 were determined 12 h after TLR3 activation. The results are expressed as mean ± SD. Significant differences were shown compared to the poly(I:C)-treated control alveolar macrophages at *p* < 0.05 (*) or *p* < 0.01 (**).

Our previous work evaluating the capacity of orally administered probiotics to modulate the respiratory innate antiviral immune response determined that alveolar macrophages are a key population involved in the beneficial effects [15]. Then, we were interested in determining whether water kefir or kefiran were capable of inducing changes in the expression of immune factors in alveolar macrophages. Mice were treated with water kefir or kefiran, and alveolar macrophages were collected one day after the last treatment, cultured, and in vitro stimulated with poly(I:C). The expression of the antiviral factors IFN-β, IFN-γ, Mx2, OAS1, RNAseL, and IFITM3 in macrophages was measured with RT-PCR (Figure 4). As expected, the expression levels of IFN-β and IFN-γ were significantly higher in alveolar macrophages from water kefir- and kefiran-treated mice compared to controls. In line with these findings, the levels of all the interferon-stimulated genes (ISGs) Mx2, OAS1, RNAseL, and IFITM3 were also significantly higher in alveolar macrophages from kefir- and water kefir-treated groups when compared to controls (Figure 4). No significant differences were observed between water kefir and kefiran treatments.

### 3.3. Water Kefir and Kefiran Improve the Resistance to RSV Infection

**Figure 5:**
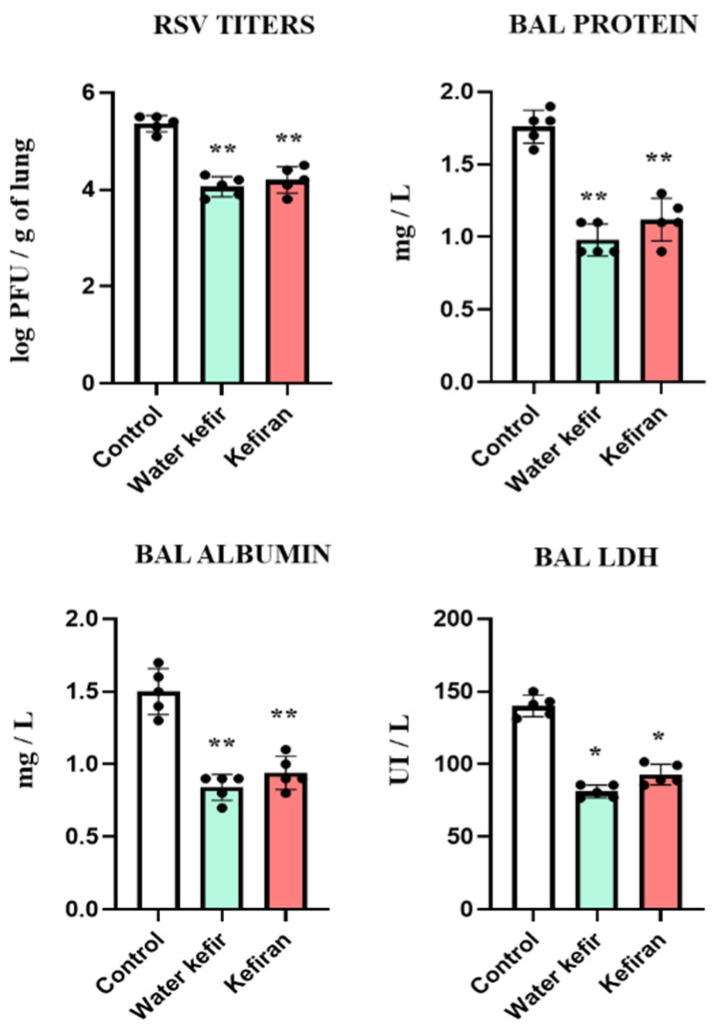
Effect of water kefir and kefiran on the resistance to RSV infection. Mice were fed water kefir or kefiran for 6 days and challenged on day 7 with RSV by the nasal route. Mice without water kefir or kefiran treatment and infected with RSV were used as control. The RSV lung titers, the concentrations of broncho-alveolar lavage (BAL) proteins and albumin, and the activity of BAL lactate dehydrogenase (LDH) were determined 2 days after RSV challenge. The results are expressed as mean ± SD. Significant differences were shown compared to the RSV-infected control group at *p* < 0.05 (*) or *p* < 0.01 (**).

Finally, we aimed to evaluate whether the changes induced by water kefir and kefiran in the respiratory antiviral immunity correlated with an improvement in the resistance to a real viral infection. Then, treated and control mice were challenged with RSV, and viral titers and lung injury parameters were determined 2 days after the infection (Figure 5). RSV efficiently infected mice, as shown by the higher viral titers in lungs and the increase in the biochemical markers of lung injury. Water kefir- and kefiran-treated mice had significantly lower viral titers and lower levels of BAL protein, albumin, and LDH in comparison to control animals (Figure 5). No differences were detected in the resistance to RSV infection when water kefir and kefiran treatments were compared.

## 4. Discussion

The effects of kefir and kefiran in the context of respiratory viral infections have not been investigated in detail before. Only one double-blind randomized controlled trial demonstrated that milk kefir can modulate the inflammatory response and reduce the severity of SARS-CoV-2 infection [10]. Water kefir and kefiran could be alternatives to milk kefir for their application in persons with lactose intolerance or milk allergy and could be incorporated into vegan diets. Using mice models, we demonstrated here for the first time that the oral administration of water kefir or kefiran can modulate the respiratory TLR3-mediated innate antiviral immunity and improve the resistance to RSV infection. In our hands, the treatment of mice with water kefir or kefiran for 6 days improved the production of IFNs and antiviral factors in the respiratory tract after the activation of the TLR3 signaling pathway, differentially modulated the balance of pro- and anti-inflammatory cytokines, reduced RSV replication, and diminished lung tissue damage.

We employed an experimental lung inflammation model using poly(I:C), an artificial TLR3 ligand, and double-stranded RNA (dsRNA) analog. In our study, poly(I:C) administration caused lung damage, accompanied by the production of pro-inflammatory mediators and the recruitment of inflammatory cells into the airways, consistently with previous findings [16,17,18]. Poly(I:C) exposure led to respiratory epithelial cell death and compromised epithelial barrier integrity, evidenced by elevated LDH activity and albumin levels in BAL fluid. Additionally, the nasal administration of poly(I:C) triggered a marked inflammatory response, with an increase in total cellularity in BAL samples, primarily driven by a significant influx of neutrophils. TLR3 activation in the respiratory tract also augmented the levels of inflammatory cytokines and chemokines. Of note, the preventive administration of water kefir or kefiran led to a notable reduction in BAL neutrophils and pro-inflammatory cytokines such as TNF-α, KC, and MCP-1 in the respiratory tract following the challenge with poly(I:C). This decrease in inflammatory cells and mediators likely contributed to the diminished lung damage observed in water kefir- and kefiran-treated mice. Additionally, water kefir and kefiran pre-treatments significantly elevated IL-10 and IL-27 levels in the respiratory tract. The rise in regulatory cytokines could play a key role in mitigating inflammatory injury and the physiological alterations caused by the poly(I:C) challenge and RSV infection. IL-10 has an important role in modulating the severity of disease during RSV infection [19,20,21]. Studies have shown that a deficiency in IL-10 during RSV infection does not impact viral load but results in substantially increased disease severity. This includes intensified weight loss, delayed recovery, a larger influx of inflammatory cells into the lungs and airways, and an enhanced release of pro-inflammatory mediators [21]. It was also reported that the immunoregulatory functions of alveolar macrophages during viral infections are of importance to maintain lung integrity. These immune cells are known to produce IL-10, particularly during the resolution phase of the infection [22,23], and also IL-6 and IL-27, which promote the maturation of Treg cells in the respiratory tract [24,25]. In fact, it was shown that the depletion of either IL-6 or IL-27 during RSV infection resulted in impaired Treg cell maturation and worsened immunopathology [25].

It was demonstrated that an overactive inflammatory response can lead to heightened immunopathology during viral respiratory infections, whereas the excessive production of anti-inflammatory cytokines may delay the clearance of the pathogen [26]. Then, maintaining a proper balance between anti-inflammatory and pro-inflammatory mediators is vital for ensuring an effective and safe antiviral immune response, and our results show that water kefir and kefiran would help to maintain that balance promoting a controlled inflammatory response that defends against infection while minimizing tissue damage.

The timely production of type I IFNs, interferon-stimulated genes (ISGs), and IFN-γ is critical for protection against RSV. Alveolar macrophages are one of the first immune cells that encounter viruses that reach the respiratory mucosa and play a key role in the defense by producing type I IFNs and IFN-γ [27]. These IFNs modulate the activity of various immune and non-immune cells in the respiratory tract, driving the expression of ISGs that aid in viral clearance [28]. Additionally, type I IFNs and IFN-γ promote the recruitment of inflammatory monocytes/macrophages further supporting the elimination of infected cells [29]. In previous studies, we observed that the oral administration of the probiotic strain *Lacticaseibacillus rhamnosus* CRL1505 led to increased levels of BAL IFN-γ following both poly(I:C) stimulation [16] and RSV infection [18]. This elevation in respiratory IFN-γ levels was attributed to a higher number of CD3^+^CD4^+^IFN-γ^+^ T cells. Orally administered CRL1505 strain also increased the levels of BAL IFN-β that correlated with higher numbers of CD11c^+^SiglecF^+^IFN-β^+^ alveolar macrophages as well as with improved expression of ISGs in this immune cell population [15]. In agreement, feeding mice with *L. gasseri* SBT2055 improved the expression of Mx1 and Oas1 in alveolar macrophages, reducing susceptibility to influenza virus (IFV) infection [30]. Similarly to these previous results, we observed here that water kefir and kefiran administration increased the levels of IFN-β and IFN-γ in the respiratory tract as well as the expression of IFNs, Mx2, OAS1, RNAseL, and IFITM3 in alveolar macrophages.

OAS1 can inhibit protein synthesis and viral growth by degrading both viral and cellular RNA, while IFITM3 blocks early events in the viral replication cycle. Both OAS1 and IFITM3 have been shown to interfere with RSV replication [29,31]. On the other hand, it was reported that RNAseL degrades viral and cellular RNA [32] conferring improved protection against RSV infection [33]. Additionally, the up-regulation of IFN-α/Mx2 levels in the respiratory tract was associated with the resistance against RSV [34]. Thus, the enhancement of these antiviral factors is consistent with the improved RSV clearance observed in water kefir- and kefiran-treated animals.

The previously published works and the data presented here cannot reveal in a conclusive manner the mechanism(s) through which water kefir or kefiran can exert beneficial effects on the respiratory tract when administered orally. Several mechanisms have been proposed to explain the effect of the intestinal microbiota on the gut–lung axis and its impact on the resistance to viral infections, which include the mobilization of immune cells from the gut to the lung, the metabolic reprogramming of respiratory innate immune cells through the production of compounds such as short-chain fatty acids (SCFAs) and the release of extracellular vesicles (EVs) (reviewed in [35]). One or more of these mechanisms could be involved in the effects of water kefir or kefiran as described below.

Research has demonstrated immunomodulatory properties of certain orally administered LAB strains that activate the common mucosal immune system, impacting not only the intestinal tract but also distant sites, such as the respiratory system [35,36]. In these studies, probiotics have been found to improve the protection against respiratory infections [35,36]. As mentioned before, both milk kefir and pasteurized milk kefir when orally administered increased the number of IgA^+^ cells in the respiratory tract as well as the phagocytic activity of pulmonary macrophages [3], while the kefiran produced by *L. kefiranofaciens* ATCC 43761 diminished NF-κB activation in the lungs induced by LPS [7]. Furthermore, BALB/c mice treated with kefiran for 7 days showed not only significant increases in the numbers of IgA^+^ plasma cells and F4/80^+^ macrophages in the small intestine lamina propria and B220^+^MHCII^high^ cells in Peyer’s patches, but also augmented F4/80^+^ macrophages in the peritoneal cavity and B220^+^MHCII^high^ cells in mesenteric lymph nodes, indicating its ability to impact immunity beyond the gut [37]. Then, it is possible to speculate that LAB strains in water kefir or kefiran would be able to stimulate the common mucosal immune system directly and enhance respiratory antiviral immunity.

On the other hand, it was reported that the oral administration of a probiotic mixture, including *L. rhamnosus* GG and *E. coli* Nissle 1917, altered the intestinal microbiota composition and metabolic profile, leading to increased production of SCFAs. These metabolic products positively affected alveolar macrophage function by enhancing the expression of IFN-β and antiviral factors, thereby increasing the resistance of adult mice to RSV infection [38]. In line with this study, it was shown that a high-fiber diet enhanced SCFA production by the intestinal microbiota of mice and that this metabolic change modulated IFN-β activity in the respiratory tract [39]. The immunological changes induced by the dietary treatment significantly increased resistance to challenge with RSV. The production of SCFAs has been described in milk kefir, with lactic acid, acetic acid, and propionic acid being the most abundant [40]. Interestingly, the EPS produced by *Lactiplantibacillus plantarum* YW11 isolated from Tibetan kefir administered to mice increased the SCFA-producing genera *Blautia*, *Butyicicoccus*, and *Allobaculum* in the intestine and consequently enhanced the SCFAs concentrations in fecal samples [41]. Metabolic changes induced by water kefir were also investigated using a short-term simulation of the colonic fermentation process [42]. In this context, water kefir was shown to increase propionate and butyrate and reduce ammonia production. Then, the presence of SCFAs in the kefir beverage and/or the stimulation of SCFA production by the intestinal microbiota could be involved in the stimulation of respiratory antiviral immunity.

It was shown that the milk kefir isolated bacteria *Lentilactobacillus kefiri* KCTC 3611 (basonym: *Lactobacillus kefir*), *Lactobacillus kefiranofaciens* KCTC 5075, and *Lactobacillus kefiranofaciens* subsp. *kefirgranum* KCTC 5086 can produce EVs [43], which are biologically active microbial structures that mediate the exchange of molecular materials between bacteria-bacteria and bacteria-host cells. EVs produced by the KCTC strains can modulate NF-κB pathway and inflammatory factor production in Caco-2 cells stimulated with TNF-α. Of note, the combination of EVs from the three strains was more efficient in modulating the inflammatory response in intestinal epithelial cells than individual EVs treatments [43]. Furthermore, the mixture of EVs reduced the inflammatory-mediated intestinal damage induced by 2,4,6-trinitrobenzene sulfonic acid (TNBS) administration in adult BALB/c mice [43]. Similarly, EVs produced by *L. kefirgranum* PRCC-1301 were shown to modulate intestinal inflammatory responses both in vitro and in vivo [44]. The treatment of Caco-2 cells with EVs from the PRCC-1301 strain significantly reduced the expression of IL-2, IL-8, and TNF-α after the challenge with dextran sulfate sodium (DSS). In addition, the effect of EVs was evaluated in adult C57BL/6 mice treated with DSS and adult IL-10^−/−^ C57BL/6 mice treated with piroxicam, which were used as acute and chronic colitis models, respectively. The study demonstrated that EVs from the PRCC-1301 strain improved the expression of ZO-1, claudin-1, and occludin proteins of colonic epithelial cells, modulated the NF-κB signaling pathway, and diminished inflammatory-mediated damage [44]. It has been shown that bacteria from the gut microbiota can produce and release EVs containing bacterial DNA, which can be shed into the bloodstream and activate extraintestinal immune cells [45]. The study found that in experimental animals, blood serum contained EVs with bacterial DNA from several bacterial phyla, including Firmicutes, Bacteroidetes, Proteobacteria, and Actinobacteriota. These EVs served as intercellular communication devices, which allow the gut microbiota to transfer its stimulus across host physiological barriers and increase the systemic antiviral immunity, thus improving the protection against herpes simplex virus type 1 and vesicular stomatitis virus [45].

An important point for future research is to elucidate whether the molecules and/or microorganisms present in water kefir exert a direct action on the mucosal immune system or whether they do so indirectly through changes in the intestinal microbiota, since there are reports describing the ability kefir to modulate gut microbiota richness and diversity in healthy volunteers [46]. Then, additional studies are necessary to characterize the precise mechanism(s) of action of water kefir and kefiran in the context of respiratory viral infections. Our results allow us to speculate that kefiran would not be a molecule involved in the immunomodulatory effects of water kefir. Despite of the fact that all the parameters evaluated in both the poly(I:C) administration and the RSV infection models were similar in the groups of animals that received water kefir and kefiran, it is well know that the main EPS from milk kefir grains is kefiran, while grain EPS from water kefir is primarily composed of α-glucans like dextran (reviewed in [2]). It is also important to verify in future investigations whether, like water kefir, milk kefir is able to improve antiviral immunity in the respiratory tract, taking into account that both foods have differences in microbiological, dry matter, protein, ash, and mineral contents. For example, it was shown that the oral administration of kefir peptides, produced during the fermentation of milk, to mice before the induction of pulmonary fibrosis with bleomycin improved pulmonary functional tests, and differentially modulated the expression of antioxidant factor-, inflammatory-, and fibrosis-related gene expressions in lung tissue [9]. Of note, full-length 16 S rRNA sequencing to study gut microbiota allowed the authors to describe differences in β-diversity in the groups of mice with or without kefir peptides treatment. *Barnesiella intestinihominis* (immunomodulatory bacterium) and *Kineothrix alysoides* (SCFA-producing bacterium) were diminished in bleomycin-treated mice compared with controls. Kefir peptides reverted these changes in gut microbiota [9]. Considering that it is unlikely that these peptides are found in water kefir, further research is necessary to elucidate which of the two foods is the most efficient in modulating respiratory immunity in general, and in particular, that associated with the defense against viruses.

## 5. Conclusions

Fortification with functional foods may offer a promising intervention to alleviate common infectious illnesses, such as viral respiratory infections, by modulating the mucosal immune system. The results presented in this work support the potential use of water kefir to improve the host’s immunity and reduce the incidence and severity of respiratory viral infections. Water kefir is a fermented drink that can be prepared at home cheaply, and therefore, it could be used as part of a massive application strategy to prevent respiratory viral infections. The microbial composition of kefir-derived beverages varies among countries, geographical regions, and the substrates; therefore, their characterization in terms of both composition and health-promoting effects is mandatory for a wider biotechnological application of kefir as an immunomodulatory functional food.

## Data Availability

The data presented in this study are available throughout the article.

## References

[1] Vieira C.P., Rosario A.I.L.S., Lelis C.A., Rekowsky B.S.S., Carvalho A.P.A., Rosário D.K.A., Elias T.A., Costa M.P., Foguel D., Conte-Junior C.A. (2021). Bioactive compounds from kefir and their potential benefits on health: A systematic review and meta-analysis. Oxid. Med. Cell Longev..

[2] Lynch K.M., Wilkinson S., Daenen L., Arendt E.K. (2021). An update on water kefir: Microbiology, composition and production. Int. J. Food Microbiol..

[3] Vinderola C.G., Duarte J., Thangavel D., Perdigon G., Farnworth E., Matar C. (2005). Distal mucosal site stimulation by kefir and duration of the immune response. Eur. J. Inflamm..

[4] Kwon O.K., Ahn K.S., Lee M.Y., Kim S.Y., Park B.Y., Kim M.K., Lee I.Y., Oh S.R., Lee H.K. (2008). Inhibitory effect of kefiran on ovalbumin-induced lung inflammation in a murine model of asthma. Arch. Pharm. Res..

[5] Griana T.P., Raras T.Y., Mintaroem K., Chozin I.N., Wilujeng C.S. (2020). Immunosuppressive activity of goat kefir in a rat model with bleomycin-induced pulmonary fibrosis. Pharmacogn. J..

[6] Chen H.L., Hung K.F., Yen C.C., Laio C.H., Wang J.L., Lan Y.W., Chong K.Y., Fan H.C., Chen C.M. (2019). Kefir peptides alleviate particulate matter <4 μm (PM_4.0_)-induced pulmonary inflammation by inhibiting the NF-κB pathway using luciferase transgenic mice. Sci. Rep..

[7] Liao C.H., Yen C.C., Chen H.L., Liu Y.H., Chen Y.H., Lan Y.W., Chen K.R., Chen W., Chen C.M. (2023). Novel kefir exopolysaccharides (KEPS) mitigate lipopolysaccharide (LPS)-induced systemic inflammation in luciferase transgenic mice through inhibition of the NF-κB pathway. Antioxidants.

[8] Lee M.Y., Ahn K.S., Kwon O.K., Kim M.J., Kim M.K., Lee I.Y., Oh S.R., Lee H.K. (2007). Anti-inflammatory and anti-allergic effects of kefir in a mouse asthma model. Immunobiology.

[9] Lan Y.W., Chen Y.C., Yen C.C., Chen H.L., Tung M.C., Fan H.C., Chen C.M. (2024). Kefir peptides mitigate bleomycin-induced pulmonary fibrosis in mice through modulating oxidative stress, inflammation and gut microbiota. Biomed. Pharmacother..

[10] Gooruee R., Pahlavani N., Hadi V., Hadi S. (2024). Evaluation of the effect of kefir supplementation on inflammatory markers and clinical and hematological indices in COVID-19 patients; a randomized double-blined clinical trial. Adv. Integr. Med..

[11] Dentice Maidana S., Ortiz Moyano R., Elean M., Imamura Y., Albarracín L., Namai F., Suda Y., Nishiyama K., Villena J., Kitazawa H. (2024). Modulation of the Toll-like receptor 3-mediated intestinal immune response by water kefir. Microbiol. Res..

[12] Medrano M., Hamet M.F., Abraham A.G., Pérez P.F. (2009). Kefiran protects Caco-2 cells from cytopathic effects induced by *Bacillus cereus* infection. Antonie Van Leeuwenhoek.

[13] Hamet M.F., Medrano M., Pérez P.F., Abraham A.G. (2016). Oral administration of kefiran exerts a bifidogenic effect on BALB/c mice intestinal microbiota. Benef. Microbes.

[14] Clua P., Kanmani P., Zelaya H., Tada A., Humayun Kober A.K.M., Salva S., Alvarez S., Kitazawa H., Villena J. (2017). Peptidoglycan from immunobiotic *Lactobacillus rhamnosus* improves resistance of infant mice to respiratory syncytial viral infection and secondary pneumococcal pneumonia. Front. Immunol..

[15] Garcia-Castillo V., Tomokiyo M., Raya Tonetti F., Islam M.A., Takahashi H., Kitazawa H., Villena J. (2020). Alveolar macrophages are key players in the modulation of the respiratory antiviral immunity induced by orally administered *Lacticaseibacillus rhamnosus* CRL1505. Front. Immunol..

[16] Villena J., Chiba E., Tomosada Y., Salva S., Marranzino G., Kitazawa H., Alvarez S. (2012). Orally administered *Lactobacillus rhamnosus* modulates the respiratory immune response triggered by the viral pathogen-associated molecular pattern poly (I: C). BMC Immunol..

[17] Stowell N.C., Seideman J., Raymond H.A., Smalley K.A., Lamb R.J., Egenolf D.D., Bugelski P.J., Murray L.A., Marsters P.A., Bunting R.A. (2009). Long-term activation of TLR3 by Poly(I:C) induces inflammation and impairs lung function in mice. Respir. Res..

[18] Chiba E., Tomosada Y., Vizoso-Pinto M.G., Takahashi T., Tsukida K., Kitazawa H., Avarez S., Villena J. (2013). Immunobiotic *Lactobacillus rhamnosus* improves resistance of infant mice against respiratory syncytial virus infection. Int. Immunopharmacol..

[19] Weiss K.A., Christiaansen A.F., Fulton R.B., Meyerholz D.K., Varga S.M. (2011). Multiple CD4+ T cell subsets produce immunomodulatory IL-10 during respiratory syncytial virus infection. J. Immunol..

[20] Sun J., Cardani A., Sharma A.K., Laubach V.E., Jack R.S., Müller W., Braciale T.J. (2011). Autocrine regulation of pulmonary inflammation by effector T-cell derived IL-10 during infection with respiratory syncytial virus. PLoS Pathog..

[21] Loebbermann J., Schnoeller C., Thornton H., Durant L., Sweeney N.P., Schuijs M., O’Garra A., Johansson C., Openshaw P.J. (2012). IL-10 regulates viral lung immunopathology during acute respiratory syncytial virus infection in mice. PLoS ONE.

[22] Bohmwald K., Espinoza J.A., Pulgar R.A., Jara E.L., Kalergis A.M. (2017). Functional impairment of mononuclear phagocyte system by the human respiratory syncytial virus. Front. Immunol..

[23] Allard B., Panariti A., Martin J.G. (2018). Alveolar macrophages in the resolution of inflammation, tissue repair, and tolerance to infection. Front. Immunol..

[24] Soroosh P., Doherty T.A., Duan W., Mehta A.K., Choi H., Adams Y.F., Mikulski Z., Khorram N., Rosenthal P., Broide D.H. (2013). Lung-resident tissue macrophages generate Foxp3+ regulatory T cells and promote airway tolerance. J. Exp. Med..

[25] Pyle C.J., Uwadiae F.I., Swieboda D.P., Harker J.A. (2017). Early IL-6 signalling promotes IL-27 dependent maturation of regulatory T cells in the lungs and resolution of viral immunopathology. PLoS Pathog..

[26] McNamara P.S., Smyth R.L. (2002). The pathogenesis of respiratory syncytial virus disease in childhood. Br. Med. Bull..

[27] Reed J.L., Brewah Y.A., Delaney T., Welliver T., Burwell T., Benjamin E., Kuta E., Kozhich A., McKinney L., Suzich J. (2008). Macrophage impairment underlies airway occlusion in primary respiratory syncytial virus bronchiolitis. J. Infect. Dis..

[28] Goritzka M., Makris S., Kausar F., Durant L.R., Pereira C., Kumagai Y., Culley F.J., Mack M., Akira S., Johansson C. (2015). Alveolar macrophage-derived type I interferons orchestrate innate immunity to RSV through recruitment of antiviral monocytes. J. Exp. Med..

[29] Goubau D., Romieu-Mourez R., Solis M., Hernandez E., Mesplède T., Lin R., Hiscott J. (2009). Transcriptional re-programming of primary macrophages reveals distinct apoptotic and anti-tumoral functions of IRF-3 and IRF-7. Eur. J. Immunol..

[30] Nakayama Y., Moriya T., Sakai F., Ikeda N., Shiozaki T., Hosoya T., Nakagawa H., Miyazaki T. (2014). Oral administration of *Lactobacillus gasseri* SBT2055 is effective for preventing influenza in mice. Sci. Rep..

[31] Schoggins J.W., MacDuff D.A., Imanaka N., Gainey M.D., Shrestha B., Eitson J.L., Mar K.B., Richardson R.B., Ratushny A.V., Litvak V. (2014). Pan-viral specificity of IFN-induced genes reveals new roles for cGAS in innate immunity. Nature.

[32] Ibsen M.S., Gad H.H., Thavachelvam K., Boesen T., Despres P., Hartmann R. (2014). The 2′-5′-oligoadenylate synthetase 3 enzyme potently synthesizes the 2′ 5′-oligoadenylates required for rnase l activation. J. Virol..

[33] Behera A.K., Kumar M., Lockey R.F., Mohapatra S.S. (2002). 2′-5′ oligoadenylate synthetase plays a critical role in interferon-γ inhibition of respiratory syncytial virus infection of human epithelial cells. J. Biol. Chem..

[34] Wen X., Mo S., Chen S., Yu G., Gao L., Chen S., Deng Y., Xie X., Zang N., Ren L. (2019). Pathogenic difference of respiratory syncytial virus infection in cotton rats of different ages. Microb. Pathog..

[35] Villena J., Kitazawa H. (2020). The Modulation of mucosal antiviral immunity by immunobiotics: Could they offer any benefit in the SARS-CoV-2 pandemic?. Front. Physiol..

[36] Villena J., Oliveira M.L., Ferreira P., Salva S., Alvarez S. (2011). Lactic acid bacteria in the prevention of pneumococcal respiratory infection: Future opportunities and challenges. Int. Immunopharmacol..

[37] Medrano M., Racedo S.M., Rolny I.S., Abraham A.G., Pérez P.F. (2011). Oral administration of kefiran induces changes in the balance of immune cells in a murine model. J. Agric. Food Chem..

[38] Ji J., Sun Q., Wang Q., Zhang H., Qin F., Wang Q., Lu S., Pang G., Lu Z. (2020). Probiotics confers protection against RSV infections by regulating gut and lung microbiotas to activate antiviral responses of alveolar macrophage. SSRN Electron. J..

[39] Antunes K.H., Fachi J.L., de Souza A.P.D. (2019). Microbiota-derived acetate protects against respiratory syncytial virus infection through a GPR43-type 1 interferon response. Nat. Commun..

[40] Ibacache-Quiroga C., González-Pizarro K., Charifeh M., Canales C., Díaz-Viciedo R., Schmachtenberg O., Dinamarca M.A. (2022). metagenomic and functional characterization of two chilean kefir beverages reveals a dairy beverage containing active enzymes, short-chain fatty acids, microbial β-amyloids, and bio-film Inhibitors. Foods.

[41] Zhang J., Zhao X., Jiang Y., Zhao W., Guo T., Cao Y., Teng J., Hao X., Zhao J., Yang Z. (2017). Antioxidant status and gut microbiota change in an aging mouse model as influenced by exopolysaccharide produced by *Lactobacillus plantarum* YW11 isolated from Tibetan kefir. J. Dairy. Sci..

[42] Calatayud M., Börner R.A., Ghyselinck J., Verstrepen L., Medts J., Abbeele P.V.D., Boulangé C.L., Priour S., Marzorati M., Damak S. (2021). Water kefir and derived pasteurized beverages modulate gut microbiota, intestinal permeability and cytokine production in vitro. Nutrients.

[43] Seo M.K., Park E.J., Ko S.Y., Choi E.W., Kim S. (2018). Therapeutic effects of kefir grain Lactobacillus-derived extracellular vesicles in mice with 2, 4, 6-trinitrobenzene sulfonic acid-induced inflammatory bowel disease. J. Dairy Sci..

[44] Kang E.A., Choi H.-I., Hong S.W., Kang S., Jegal H.-Y., Choi E.W., Park B.-S., Kim J.S. (2020). Extracellular vesicles derived from kefir grain Lactobacillus ameliorate intestinal inflammation via regulation of proinflammatory pathway and tight junction integrity. Biomedicines.

[45] Erttmann S.F., Swacha P., Gekara N.O. (2022). The gut microbiota prime systemic antiviral immunity via the cGAS-STING-IFN-I axis. Immunity.

[46] Sepp E., Smidt I., Štšepetova J., Rööp T., Hütt P., Rätsep M., Mikelsaar M. (2018). The effect of *Lactobacillus fermentum* ME-3 on the intestinal microbiota and urine polyamines content: A double-blind placebo-controlled pilot trial. J. Funct. Foods..

